# Development and Application of Two Inducible Expression Systems for Streptococcus suis

**DOI:** 10.1128/spectrum.00363-22

**Published:** 2022-06-27

**Authors:** Liangsheng Zhang, Wenjin Zou, Minghui Ni, Qiao Hu, Lelin Zhao, Xia Liao, Qi Huang, Rui Zhou

**Affiliations:** a State Key Laboratory of Agricultural Microbiology, College of Veterinary Medicine, Huazhong Agricultural University, Wuhan, China; b Cooperative Innovation Center of Sustainable Pig Production, Wuhan, China; c International Research Center for Animal Disease, Ministry of Science and Technology of China, Wuhan, China; d The HZAU-HVSEN Institute, Wuhan, China; University of Manitoba

**Keywords:** *Streptococcus suis*, genetic tool, inducible expression system, subcellular localization, conditional gene knockout

## Abstract

Streptococcus suis is an important zoonotic bacterial pathogen posing a threat to the pig industry as well as public health, for which the mechanisms of growth and cell division remain largely unknown. Developing convenient genetic tools that can achieve strictly controlled gene expression is of great value for investigating these fundamental physiological processes of S. suis. In this study, we first identified three strong constitutive promoters, P_g_, P_t_, and P_e_, in S. suis. Promoter P_g_ was used to drive the expression of repressor genes *tetR* and *lacI*, and the operator sequences were added within promoters P_t_ and P_e_. By optimizing the insertion sites of the operator sequence, we successfully constructed an anhydrotetracycline (ATc)-inducible expression system and an isopropyl-β-d-thiogalactopyranoside (IPTG)-inducible expression system in S. suis. We showed that these two systems provided inducer-concentration- and induction-time-dependent expression of the reporter gene. By using these tools, we investigated the subcellular localization of a key cell division protein, FtsZ, which showed that it could be correctly localized to the midcell region. In addition, we constructed a conditional knockout strain for the *glmS* gene, which is an essential gene, and showed that our ATc-inducible promoter could provide strictly controlled expression of *glmS* in *trans*, suggesting that our inducible expression systems can be used for deletion of essential genes in S. suis. Therefore, for the first time we developed two inducible expression systems in S. suis and showed their applications in the study of an important cell division protein and an essential gene. These genetic tools will further facilitate the functional study of other important genes of S. suis.

**IMPORTANCE** Streptococcus suis is an important zoonotic bacterial pathogen. Studying the mechanisms of cell growth and division is important for the identification of novel antimicrobial drug targets. Inducible expression systems can provide strictly controlled expression of the protein of interest and are useful tools to study the functions of physiologically important proteins. However, there is a lack of convenient genetic tools that can achieve inducible protein expression in S. suis. In this study, we developed two (ATc-inducible and IPTG-inducible) inducible expression systems and showed their applications in a subcellular localization study of a cell division protein and the construction of conditional knockout of essential genes in S. suis. These systems will be useful for functional studies of important proteins of S. suis.

## INTRODUCTION

Streptococcus suis is an important zoonotic pathogen causing meningitis, arthritis, endocarditis, pneumonia, and septicemia in pigs; it can also lead to streptococcal toxic shock-like syndrome (STSLS) in humans, with very high mortality rates (1–3). Performing studies regarding the etiology as well as the mechanisms of pathogenesis of S. suis is critical for developing novel strategies to prevent and control the disease. To date, extensive studies have been carried out and advances have been made regarding the pathobiology of this important pathogen (4–7). However, it needs to be pointed out that convenient genetic tools for manipulating the genome of S. suis are a prerequisite for these studies. The most commonly used genetic tools for S. suis have been developed by D. Takamatsu and colleagues, who established a set of genetic tool systems including the temperature-sensitive (Ts) suicide vectors pSET4s, pSET5s, and pSET6s for gene knockout and the replicating vectors pSET1, pSET2, and pSET3 for gene expression (8, 9). However, there is still a lack of genetic tools that can provide inducible expression of proteins in S. suis.

Bacteria replicate through binary fission, which involves DNA replication and segregation, cell growth, and cell division (10). These are the most fundamental and important physiological processes, which are attracting antimicrobial drug targets (11–13). As an oval coccus, S. suis undertakes a usual pattern of elongation and division, the molecular mechanisms of which are not yet fully understood. There are several dozen proteins that participate in cell growth and division, and many of them are reported to be essential and need precise regulation (10, 14). FtsZ is one of the most important proteins during bacterial cell division and serves as a scaffold to recruit other cell division proteins (15). Proper subcellular localization of FtsZ ensures the subsequent assembly of other cell division proteins (16). GlmS is a glutamine-fructose-6-phosphate aminotransferase involved in the conversion of fructose-6-phosphate (Fru-6P) to glucosamine-6-phosphate (GlcN-6P), which is an initial substrate for peptidoglycan synthesis (17, 18). In Streptococcus mutans, *glmS* is essential when cells are cultured in tryptic soy broth (TSB), while a *glmS* deletion mutant could survive in the presence of *N*-acetylglucosamine (GlcNAc) (19). Considering the importance and essentiality of the genes related to cell growth and division, achieving regulated or induced expression and conditional gene knockout is critical to elucidating the functions of these genes.

Inducible expression systems usually act at the level of transcription initiation by repressor proteins responding to small molecules. TetR and LacI are the most commonly used repressor proteins, for which the repression effect can be relieved by the presence of anhydrotetracycline (ATc) and isopropyl-β-d-thiogalactopyranoside (IPTG), respectively (20, 21). TetR can bind to the operator sequence *tetO*, which inhibits the expression of the tetracycline resistance gene in the absence of tetracycline. Once tetracycline is present, the binding affinity is significantly reduced, leading to the expression of the resistance gene (22, 23). The IPTG-inducible expression system comes from the lactose operon of Escherichia coli. When the inducer is present, the *lac* repressor binds to the inducer and reduces the binding to the operator sequence (20).

Here, we identified three constitutive promoters, P_g_, P_t_, and P_e_, from the S. suis genome that can be expressed steadily under different culture conditions. Based on them, we successfully developed two inducible expression systems in which the expression can be induced by ATc or IPTG. We demonstrate that the systems are useful tools that can be used to study the subcellular localization of cell division proteins and to construct conditional knockout mutants of essential genes. For the first time, we provide these genetic tools, which will greatly facilitate studies concerning essential genes or genes whose expression needs fine regulation in such an important zoonotic bacterial pathogen.

## RESULTS

### Construction of a stable expression system.

**FIG 1:**
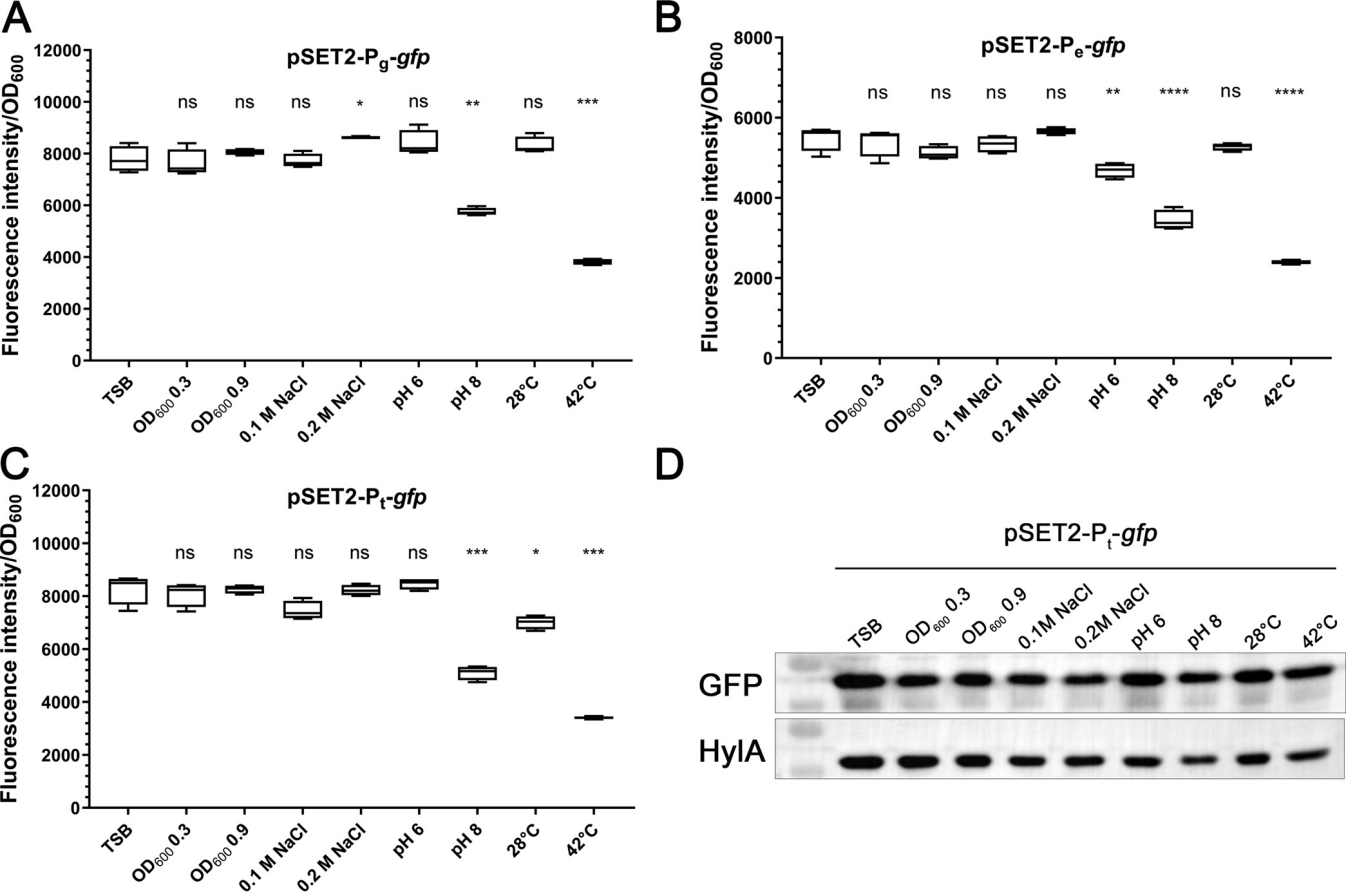
(A to C) Evaluation of the expression of the constitutive promoters. S. suis cells harboring pSET2-P_g_-*gfp* (A), pSET2-P_e_-*gfp* (B), and pSET2-P_t_-*gfp* (C) were grown under different conditions based on TSB or to different growth stages. The pH was adjusted using HCl or NaOH. The cells were harvested and washed with PBS, and the fluorescence intensity was measured using a spectrometer. Statistical analysis was performed using the unpaired Student's *t* test to compare the fluorescence intensity of the cells cultured under each indicated growth condition with that of the cells cultured in TSB. *, *P* < 0. 05; **, *P* < 0. 01; ***, *P* < 0.001; ****, *P* < 0.0001; ns, not significant. (D) Detection of GFP expression by Western blotting. S. suis cells harboring plasmid pSET2-P_t_-*gfp* and grown under different culture conditions were collected, washed with PBS, and lysed, followed by immunoblot analysis using anti-GFP or an-HylA antibody. The HylA antibody was used as a control.

Constitutive promoters can be used to engineer efficient inducible promoters (24). In S. suis, we selected three promoters, P_g_, P_e_, and P_t_, from the genome that drive the expression of the housekeeping gene *gapdH*, which has been used as an internal reference for quantitative reverse transcription-PCR (RT-qPCR) (25), the protein enolase, which has been used as an internal reference for Western blotting, and the elongation factor Tu, which has been reported to be constitutively expressed in other bacteria (26). The *gfp* gene was cloned downstream of the promoters to test their activity, resulting in three plasmids, i.e., pSET2-P_g_-*gfp*, pSET2-P_e_-*gfp*, and pSET2-P_t_-*gfp*. The plasmids were introduced individually into S. suis, and the cells were cultured for different periods of time (optical density at 600 nm [OD_600_] of 0.3 to 0.9), at a pH of 6 or 8, or at different temperatures (28°C or 42°C). Fluorescence intensity results indicated that the expression of promoters P_g_, P_t_, and P_e_ was relatively stable under most conditions except for growth at 42°C or at pH 8 (Fig. 1). Since previous studies showed that high temperatures and alkaline medium can interfere with the fluorescence intensity of green fluorescent protein (GFP) (26, 27), we further verified the expression level by Western blotting, which showed that GFP was expressed at similar levels under different growth conditions (Fig. 1D). These data suggest that promoters P_g_, P_t_, and P_e_ are constitutive promoters in S. suis.

### Construction of ATc- and IPTG-inducible expression systems.

**FIG 2:**
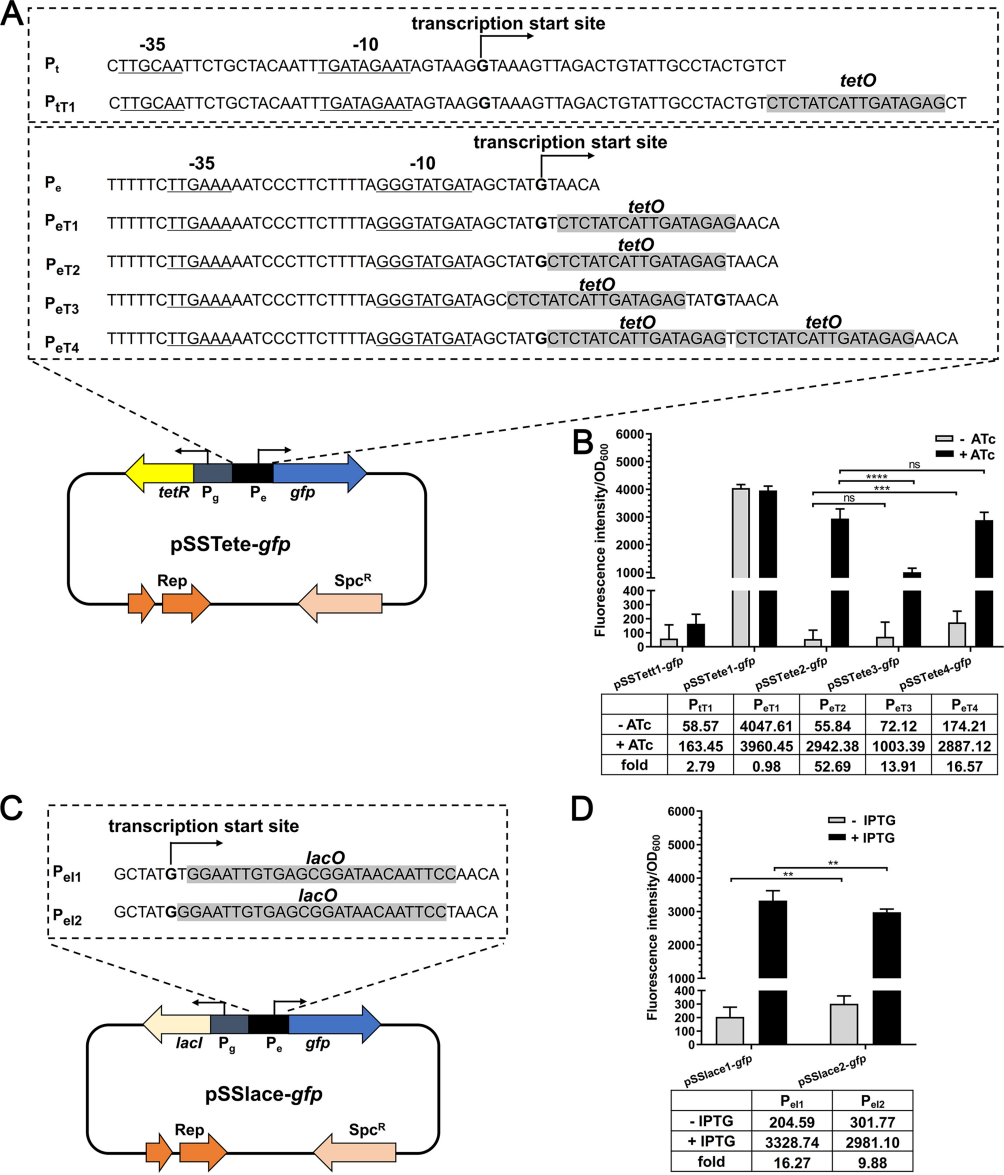
Construction and evaluation of the ATc- and IPTG-inducible expression systems. (A) Characterization of ATc-inducible promoters. The putative −10 and −35 regions and the transcription start site of the promoters were analyzed by using Softberry BPROM software. The expression of *tetR* was driven by P_g_, and that of *gfp* was driven by P_e_. The operator sequence was inserted into different positions of P_e_. (B) Detection of the expression of the ATc-inducible expression systems. S. suis cells harboring the indicated plasmid were grown to the mid-log phase and induced with or without 200 ng/mL ATc for 60 min at 37°C. The cells were harvested and washed with PBS, and the fluorescence intensity was measured using a spectrometer. − ATc, average expression level in the absence of inducer; + ATc, average expression level in the presence of inducer; fold, fold change with ATc, compared with the value without ATc. Statistical analysis was performed using the unpaired Student's *t* test to compare the activity of promoters P_eT2_, P_eT3_, and P_eT4_ with or without 200 ng/mL ATc. **, *P* < 0. 01; ***, *P* < 0.001; ****, *P* < 0.0001; ns, not significant. The data are presented as the mean ± standard deviation. (C) Characterization of IPTG-inducible promoters. The expression of *lacI* was driven by P_g_, and that of *gfp* was driven by P_e_. The operator sequence was inserted into different positions of P_e_. (D) Detection of the expression of the IPTG- inducible expression systems. S. suis cells harboring the indicated plasmid were grown to the mid-log phase and induced with or without 0.2 mM IPTG for 60 min at 37°C. – IPTG, average expression level in the absence of inducer; + ITPG, average expression level in the presence of inducer; fold, fold change with IPTG, compared with the value without IPTG. Statistical analysis was performed using the unpaired Student's *t* test to compare the activity of promoters P_eI1_ and P_eI2_ with or without 0.2 mM IPTG.

An inducible expression system harbors the element for expression of a repressor protein and a promoter inserted with an operator sequence. To construct an ATc-inducible expression system, the repressor gene *tetR* was driven by promoter P_g_, and the operator sequence was inserted into promoters P_t_ and P_e_. The putative −10 and −35 regions and the transcription start site of the promoters were analyzed by using Softberry BPROM software (28) (Fig. 2A). First, the operator sequence *tetO* was inserted at the +27 position of promoter P_t_ and at the +2 position of promoter P_e_, which were subsequently cloned into pSET2 vector, together with the repressor cassette, resulting in two plasmids, pSSTett1-*gfp* and pSSTete1-*gfp*, respectively (Fig. 2A). Fluorescence intensity results showed that the cells harboring pSSTett1-*gfp* did not show GFP expression even in the presence of inducer ATc (Fig. 2B), suggesting that the insertion of the operator sequence disrupted the promoter P_t_. In contrast, the cells harboring pSSTete1-*gfp* displayed strong expression of GFP. However, the expression was observed even in the absence of ATc, suggesting uncontrolled expression. Therefore, we next inserted the operator sequence at different positions within promoter P_e_ and also tried to insert two operator sequences, resulting in three plasmids, pSSTete2-*gfp*, pSSTete3-*gfp*, and pSSTete4-*gfp* (Fig. 2B). It is shown in Fig. 2B that plasmid pSSTete2-*gfp* had higher expression of GFP in the presence of ATc and the GFP expression was tightly repressed in the absence of inducer. Therefore, pSSTete2-*gfp* was successfully constructed as an ATc-inducible expression plasmid.

Using the same strategy to construct an IPTG-inducible system, promoter P_g_ was used to drive the expression of the repressor LacI. The operator sequence *lacO* was inserted at the +2 or +1 position of promoter P_e_, resulting in two IPTG-inducible plasmids, pSSlace1-*gfp* and pSSlace2-*gfp*, respectively (Fig. 2C). Fluorescence intensity results showed that, compared with the cells cultured in the absence of IPTG, the presence of the inducer significantly enhanced the expression of GFP, indicating an effective inducing effect (Fig. 2D). We also observed that the expression of GFP by pSSlace1-*gfp* was significantly higher than that by pSSlace2-*gfp* in the presence of IPTG (Fig. 2D). However, it should be noted that basal expression was observed for both of the plasmids when no IPTG was added (Fig. 2D). Therefore, the IPTG-inducible expression plasmid pSSlace1-*gfp* was successfully constructed.

### Regulatory capacities of the inducible expression systems.

**FIG 3:**
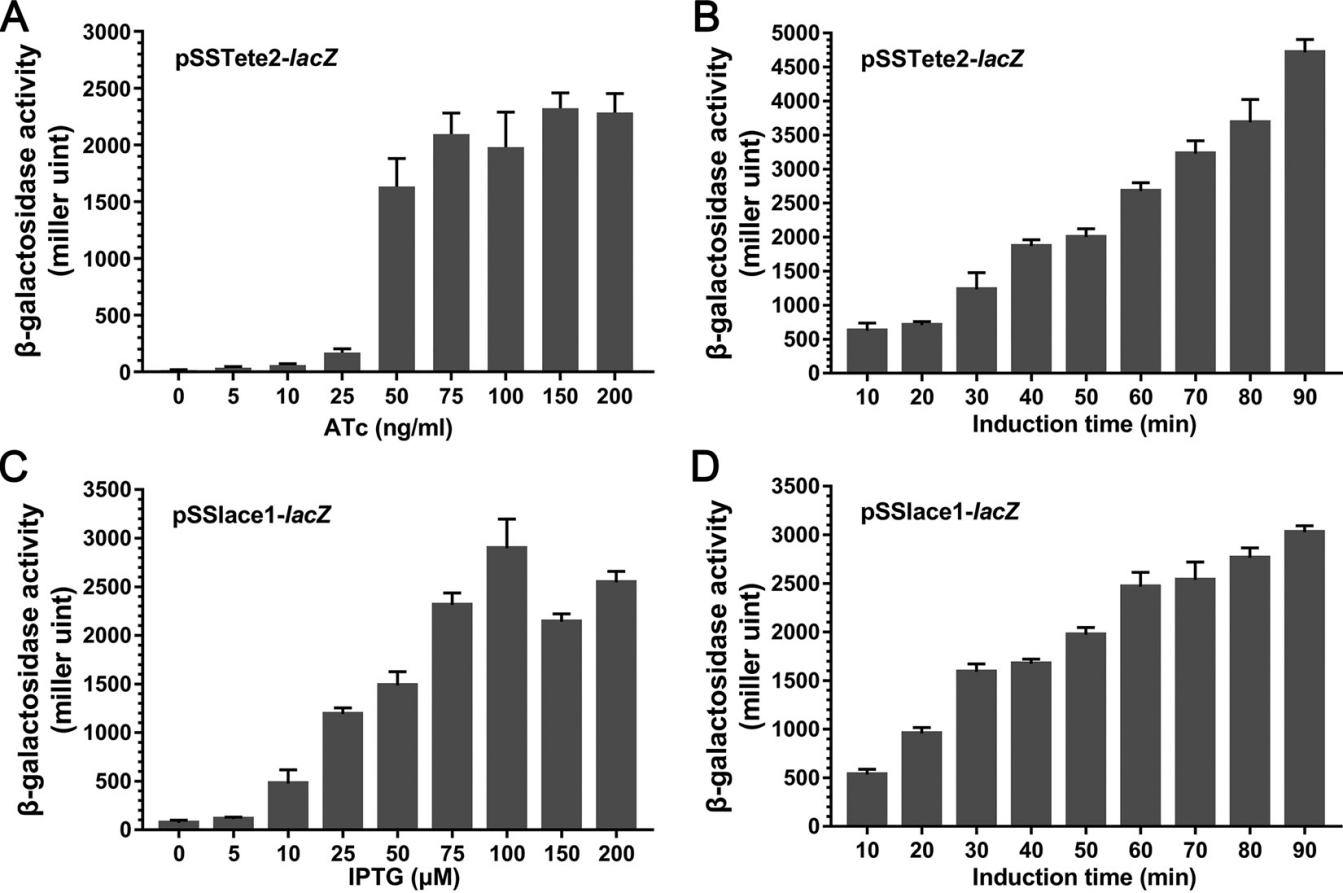
Regulatory capacities of the inducible expression systems. (A) Dose-dependent expression of pSSTete2-*lacZ*. S. suis cells harboring pSSTete2-*lacZ* plasmid were grown to the mid-log phase and induced with a range of different ATc concentrations for 70 min at 37°C. The cells were harvested, and the β-galactosidase activity was determined as described in Materials and Methods. The data are presented as the mean ± standard deviation. (B) Time-dependent expression of pSSTete2-*lacZ*. S. suis cells harboring pSSTete2-*lacZ* plasmid were grown to the mid-log phase and induced for different times in the presence of 150 ng/mL ATc at 37°C. The cells were harvested, and the β-galactosidase activity was determined as described in Materials and Methods. The data are presented as the mean ± standard deviation. (C) Dose-dependent expression of pSSlace1-*lacZ*. S. suis cells harboring pSSlace1-*lacZ* plasmid were grown to the mid-log phase and induced with a range of different IPTG concentrations for 70 min at 37°C. The cells were harvested, and the β-galactosidase activity was determined as described in Materials and Methods. The data are presented as the mean ± standard deviation. (D) Time-dependent expression of pSSlace1-*lacZ*. S. suis cells harboring pSSlace1-*lacZ* plasmid were grown to the mid-log phase and induced for different times in the presence of 100 μM/mL IPTG at 37°C. The cells were harvested, and the β-galactosidase activity was determined as described in Materials and Methods. The data are presented as the mean ± standard deviation.

We used another reporter gene, *lacZ*, to quantify the regulatory capacities of the inducible expression systems; *lacZ* was cloned into plasmids pSSTete2-*gfp* and pSSlace1-*gfp* to replace *gfp*. The resulting plasmids were introduced into S. suis and the expression of *lacZ* was quantified by measuring the β-galactosidase activity in the presence of different concentrations of inducers or with different induction times. For pSSTete2-*lacZ*, it is shown in Fig. 3A that, in the absence of ATc, β-galactosidase activity was barely detected (Fig. 3A). With the increase of the concentration of ATc, the expression of *lacZ* increased significantly, and ATc at 150 ng/mL could already give very high expression of the reporter gene (Fig. 3A). The expression of *lacZ* at a given concentration of inducer (150 ng/mL) but with different induction times showed that the expression of *lacZ* increased with the increase in induction time (Fig. 3B). For pSSlace1-*lacZ*, we also observed increased expression of *lacZ* in an inducer concentration-dependent manner, and it was fully induced with the presence of 0.1 mM IPTG (Fig. 3C). Also, prolonged induction time significantly promoted the expression of the reporter. These results suggested that plasmids pSSTete2-*lacZ* and pSSlace1-*lacZ* were both effective inducible expression systems in which the ATc-inducible plasmid pSSTete2-*lacZ* showed less basal expression.

### Determination of the subcellular localization of FtsZ in S. suis using the inducible expression system.

**FIG 4:**
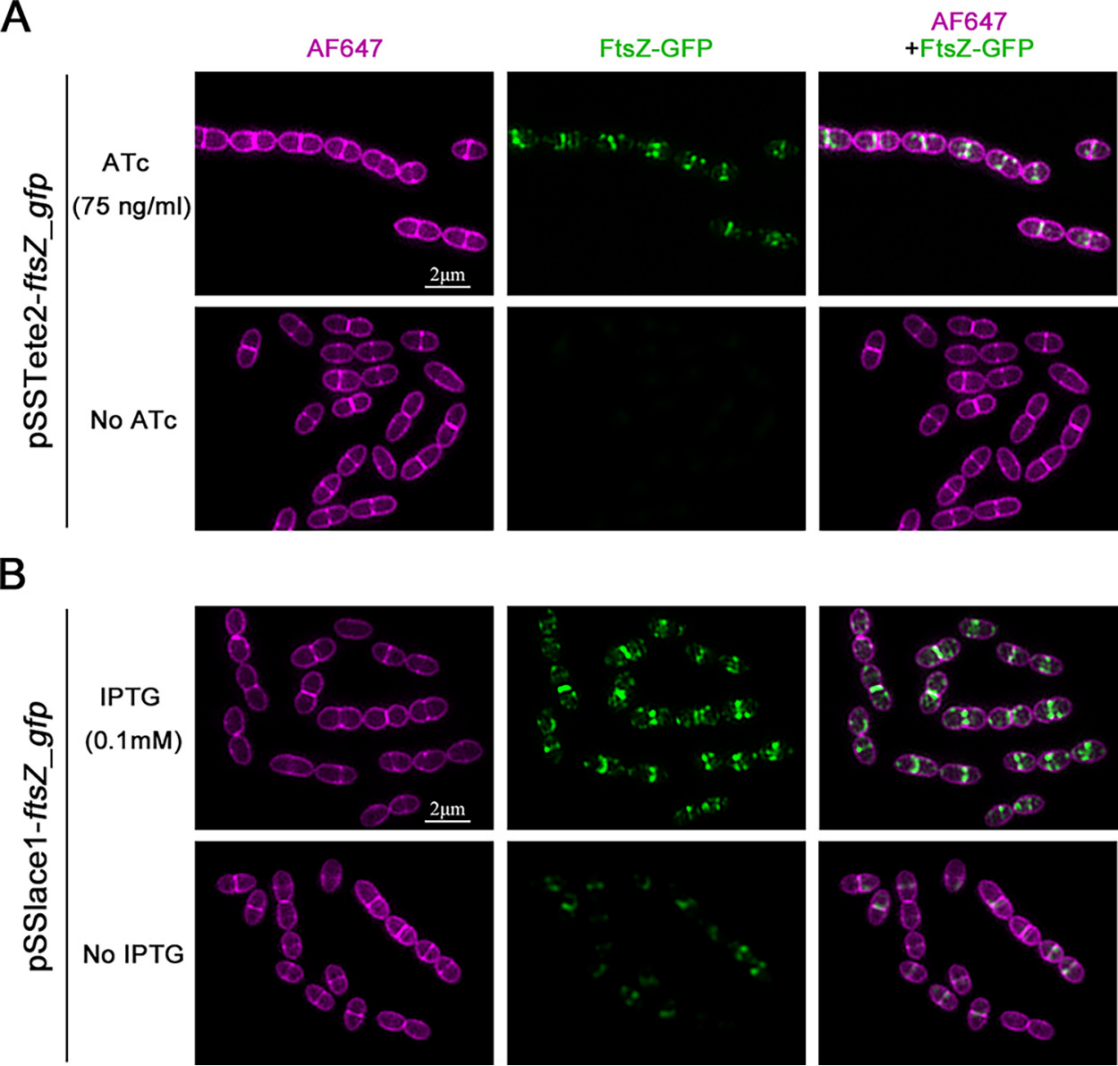
Determination of subcellular localization of FtsZ *in* S. suis using the inducible expression system. (A) Determination of subcellular localization of FtsZ using pSSTete2-*ftsZ*_*gfp*. S. suis cells harboring pSSTete2-*ftsZ*_*gfp* were grown to the mid-log phase, followed by another 15-min incubation with or without ATc induction at 75 ng/mL. The cells were harvested, washed, stained with AF647 dye, and imaged using the NIS-Elements microscope (Nikon). (B) Determination of subcellular localization of FtsZ using pSSlace1-*ftsZ*_*gfp*. S. suis cells harboring pSSlace1-*ftsZ*_*gfp* were grown to the mid-log phase, followed by another 15-min incubation with or without IPTG induction at 0.2 mM. The cells were harvested, washed, stained with AF647 dye, and imaged using the NIS-Elements microscope (Nikon).

Cell division is a very important process for bacteria. Proteins involved in this process need to be correctly localized to the destined subcellular locations to exert their functions, and it is important to be able to characterize the subcellular localization of these proteins. Therefore, we used the above-established inducible expression system to study the subcellular localization of an essential cell division protein, FtsZ, in S. suis. A *ftsZ*-*gfp* fusion was cloned into pSSTete2-*gfp* and pSSlace1-*gfp* to replace *gfp*, resulting in plasmids pSSTete2-*ftsZ_gfp* and pSSlace1-*ftsZ_gfp*, respectively. The plasmids were transformed into S. suis, and the cells were cultured in the presence of inducer, followed by examination by fluorescence microscopy. It is shown in Fig. 4 that, by using either pSSTete2-*ftsZ_gfp* or pSSlace1-*ftsZ_gfp* in the presence of inducer, a clear FtsZ-GFP signal that was localized at the middle of the cells was observed (Fig. 4), which was consistent with the localization pattern of FtsZ reported previously for S. suis (29, 30). In addition, by using plasmid pSSlace1-*ftsZ_gfp*, a weak FtsZ-GFP signal was observed even without induction, indicating basal expression, which was consistent with the observations above. These results suggest that the inducible expression systems can be used for subcellular localization studies for cell division proteins.

### Use of the inducible expression system for conditional gene knockout in S. suis.

**FIG 5:**
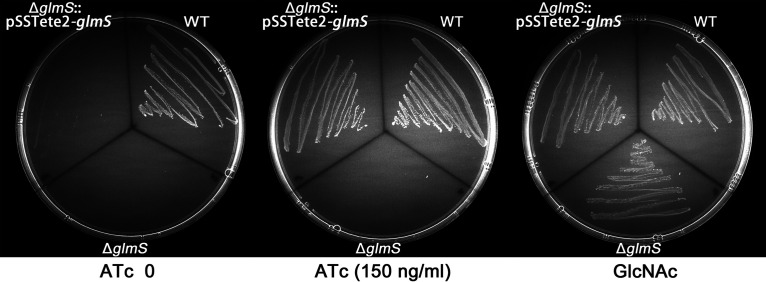
Use of pSSTete2-*glmS* for efficient gene complementation and gene depletion. S. suis wild-type (WT), Δ*glmS*, and Δ*glmS* strains harboring the pSSTete2-*glmS* plasmid were streaked on TSA plates supplemented with 10 mM GlcNAc or 150 ng/mL ATc as indicated. The plates were incubated at 37°C overnight.

It is difficult to study the functions of essential genes because knockout mutants cannot be obtained. By using an inducible expression system, however, essential gene mutants can be constructed (31). In S. suis, there were so far no available tools to construct knockout mutants for essential genes. The *glmS* gene is an essential gene that encodes glutamine-fructose-6-phosphate aminotransferase, which is involved in the conversion of Fru-6P to GlcN-6P (17). A previous study reported that the *glmS* deletion mutant was viable in the presence of GlcNAc (19) but lethal under normal growth conditions. Therefore, we constructed an S. suis *glmS* gene deletion mutant (Δ*glmS*) in the presence of GlcNAc. It was shown that, consistent with the previous finding, the S. suis Δ*glmS* mutant could not grow in the absence of GlcNAc (Fig. 5). We next constructed an inducible expression plasmid encoding *glmS*, pSSTete2-*glmS*, which was transformed into the S. suis Δ*glmS* strain. As shown in Fig. 5, the S. suis Δ*glmS* strain and the Δ*glmS* strain harboring pSSTete2-*glmS* but without ATc induction could not grow on tryptic soy agar (TSA) plates in the absence of GlcNAc but could grow when GlcNAc was supplemented (Fig. 5). However, it was seen that the Δ*glmS* strain harboring pSSTete2-*glmS* could grow in the presence of 150 ng/mL ATc on TSA plates without GlcNAc (Fig. 5). These results suggest that pSSTete2-*glmS* can provide strict control of expression that is inducer dependent. Therefore, this inducible plasmid system enables conditional deletion of essential genes.

## DISCUSSION

Bacterial resistance to antibiotics has become a serious threat to public health, and it is urgent to develop new drugs to deal with antimicrobial resistance. Common antibiotic drug targets are the protein machineries of fundamental biological processes, such as DNA and RNA synthesis, cell division, bacterial metabolism, and protein synthesis (11–13). Most of the coding genes are essential and cannot be knocked out using the traditional gene deletion approaches. This makes it difficult to study the functions of these essential genes. Unlike model microorganisms, S. suis is an oval coccus in which the mechanism of cell growth and division is distinct from that of typical bacilli or cocci (32). Here, we successfully developed two (ATc-inducible and IPTG-inducible) inducible expression systems that can be used for regulated expression and conditional knockout of essential genes in the important zoonotic pathogen S. suis.

Bacterial gene expression is influenced by a variety of environmental stimuli, including nutrient availability, ions, and temperature, on the basis of which several inducible expression systems have been developed, for example, the IPTG-inducible system (21), the ATc-inducible system (20), the arabinose-inducible system (33), and the Zn-inducible system (31). An inducible expression system usually includes a repressor protein that is normally expressed and binds the operator sequence within the promoter region, leading to repression of the target gene. Once the inducer is present, the binding between the repressor protein and the operator sequence is relieved and the target gene starts to be expressed. Therefore, to construct inducible expression systems in S. suis, we first identified three strong constitutive promoters, P_g_, P_t_, and P_e_. To ensure efficient gene repression in the absence of inducer, the relatively stronger promoter P_g_ was used to drive the expression of the repressor gene. However, when the operator sequence was inserted into P_t_, its activity was disrupted. Therefore, we then optimized the insertion site of the operator sequence with promoter P_e_, and two inducible expression plasmids, pSSTete2-*gfp* (ATc inducible) and pSSlace1-*gfp* (IPTG inducible), were successfully constructed in S. suis.

We next evaluated the regulatory capacity of these two inducible expression systems. Both of the plasmids showed inducer-concentration-dependent and induction-time-dependent expression of the reporter gene, indicating successful construction of the expression systems. However, it was noted that, compared with pSSTete2-*lacZ*, pSSlace1-*lacZ* showed higher basal expression in the absence of inducer. This is consistent with previous findings that the *lac* promoter shows leaky expression even in the presence of the repressor LacI (34). This can be resolved by increasing the expression of the repressor or by inserting more operator sequences into the promoter.

S. suis is an important zoonotic pathogen posing a severe threat to public health. To date, there are still many unknowns regarding the fundamental biology and pathogenesis of this pathogen that deserve deep investigation. Unfortunately, very limited genetic tools have been developed and no inducible expression systems are available for S. suis. The easiest way to develop inducible expression systems is to directly clone an inducible expression system that has already been developed from other bacteria. For example, the tetracycline-inducible promoter P_xyl/tet_ from Staphylococcus aureus has been applied to Streptococcus pneumoniae and Streptococcus agalactiae (35, 36). However, further optimizations are still needed; otherwise, they may not be able to provide ideal expression levels (35, 37). Another way to develop novel inducible expression systems is to screen the natural inducible expression systems in the bacterial genome by adding different inducers. By using this strategy, the ComS peptide-regulated system (38), the zinc-inducible promoter P_czcD_ (31), the maltose-inducible promoter P_M_ (39), and the fucose-inducible promoter P_fcsK_ in S. pneumoniae (40) were developed.

Bacterial cell division is one of the most fundamental physiological processes. Proteins involved in this process need precise temporal and spatial regulation. Examining the subcellular localizations of important cell division proteins is critical to studying the details of the cell division process. FtsZ plays a central role during cell division; it forms a ring structure and orchestrates cell division. Therefore, it is important to investigate its subcellular localization. However, it has been reported that overexpression of FtsZ in bacteria results in abnormal cell division (41, 42). Therefore, care is needed when performing localization studies with FtsZ. Utilizing the inducible expression systems we established in this study, it is feasible to achieve fine control of FtsZ expression, and our results showed that FtsZ-GFP expressed from both of the plasmids was localized correctly to the midcell region, suggesting that our inducible expression systems provide powerful tools for functional and localization studies of cell division proteins in S. suis.

Many important proteins involved in cell growth and division are essential, and null mutants cannot be obtained using normal genetic tools, which hinders the functional study of these proteins. Recently, however, a conditional knockout strategy that can be used to construct deletion mutants of essential genes has been developed (43). In this study, by using the inducible expression system, we showed that, in the absence of inducer, pSSTete2-*glmS* barely expressed *glmS*, indicating that the growth of S. suis Δ*glmS* needs GlcNAc. Once induced, expression from pSSTete2-*glmS* complemented the need of S. suis Δ*glmS* for GlcNAc. Therefore, our inducible expression systems can be exploited to construct gene deletion mutants of essential genes in S. suis.

In summary, we successfully constructed two inducible expression systems in S. suis. We showed their practical applications in the subcellular localization study of the critical cell division protein FtsZ, and we demonstrated their potential use in the construction of null mutants of essential genes. These systems provide useful tools that will further facilitate the functional study of important proteins of S. suis.

## MATERIALS AND METHODS

### Bacterial strains and growth conditions.

The bacterial strains and plasmids used in this study are listed in Table 1. S. suis strains in this study are derivatives of S. suis SC19, a highly virulent serotype 2 strain that was isolated from a diseased pig during the S. suis outbreak in 2005 in the Sichuan Province of China; its genome sequence GenBank accession number is NZ_CP020863.1 (44). S. suis cells were grown at 37°C with shaking at 180 rpm in TSB or on TSA (Difco, France) plates containing 5% inactivated newborn bovine serum (Sijiqing Biotech, Hangzhou, China). Spectinomycin was used at a final concentration of 100 μg/mL when needed. E. coli strain MC1061 was used for plasmid propagation (9). E. coli was cultured in Luria-Bertani (LB) broth (Difco) or on LB agar plates at 37°C.

**TABLE 1:** Bacterial strains and plasmids used in the present study

Bacterial strain or plasmid	Description^*a*^	Reference or source
Bacterial strains		
E. coli MC1061	Cloning host for recombinant vector pSET2	9
S. suis SC19	S. suis serotype 2, wild type	44
Δ*glmS*	S. suis SC19 *glmS* deletion mutant	This study
Δ*glmS*::pSSTete2-*glmS*	Δ*glmS* strain carrying recombinant plasmid pSSTete2-*glmS*	This study
Plasmids		
pSET4s	S. suis Ts suicide vector; Spc^r^	8
pSET2	S. suis expression vector; Spc^r^	9
pET28a	Expression vector; Kan^r^	Novagen
pRAB11	E. coli/S. aureus shuttle vector, containing a tetracycline-inducible promoter; Chl^r^	37
pSET2-P_g_-*gfp*	pSET2 with P_g_- *gfp* fusion cloned at BamHI site; Spc^r^	This study
pSET2-P_t_-*gfp*	pSET2 with P_t_- *gfp* fusion cloned at BamHI site; Spc^r^	This study
pSET2-P_e_-*gfp*	pSET2 with P_e_- *gfp* fusion cloned at BamHI site; Spc^r^	This study
pSSTett1-*gfp*	pSET2 with P_g_- *tetR* fusion and P_tT1_- *gfp* fusion; Spc^r^	This study
pSSTete1-*gfp*	pSET2 with P_g_- *tetR* fusion and P_eT1_- *gfp* fusion; Spc^r^	This study
pSSTete2-*gfp*	pSET2 with P_g_- *tetR* fusion and P_eT2_- *gfp* fusion; Spc^r^	This study
pSSTete3-*gfp*	pSET2 with P_g_- *tetR* fusion and P_eT3_- *gfp* fusion; Spc^r^	This study
pSSTete4-*gfp*	pSET2 with P_g_- *tetR* fusion and P_eT4_- *gfp* fusion; Spc^r^	This study
pSSlace1-*gfp*	pSET2 with P_g_- *tetR* fusion and P_eI1_- *gfp* fusion; Spc^r^	This study
pSSlace2-*gfp*	pSET2 with P_g_- *tetR* fusion and P_eI2_- *gfp* fusion; Spc^r^	This study
pSSTete2-*lacZ*	pSET2 with P_g_- *tetR* fusion and P_eT2_- *lacZ* fusion; Spc^r^	This study
pSSlace1-*lacZ*	pSET2 with P_g_- *tetR* fusion and P_eI1_- *lacZ* fusion; Spc^r^	This study
pSSTete2-*ftsZ*_*gfp*	pSET2 with P_g_- *tetR* fusion and P_eT2_- ftsZ_*gfp* fusion; Spc^r^	This study
pSSlace1-*ftsZ*_*gfp*	pSET2 with P_g_- *tetR* fusion and P_eI1_- ftsZ_*gfp* fusion; Spc^r^	This study
pSET4S-*glmS*	Derived from pSET4s for deleting *glmS* in SC-19; Spc^r^	This study
pSSTete2-*glmS*	pSET2 with P_g_- *tetR* fusion and P_eT2_- *glmS* fusion; Spc^r^	This study

a Spc^r^, spectinomycin resistance; Kan^r^, kanamycin resistance; Chl^r^, chloramphenicol resistance.

### Cloning procedures and transformation.

The promoters P_g_, P_t_, and P_e_ are the 160-bp fragment upstream of the gene *gapdH*, the 160-bp fragment upstream of the gene *tufA*, and the 118-bp fragment upstream of the gene *eno* (encoding enolase), respectively. These promoter fragments were amplified by PCR using P_g_-F/P_g_-R, P_t_-F/P_t_-R, and P_e_-F/P_e_-R, respectively (Table 2). The GFP coding sequence (*gfp*) was amplified from pMIDG301, which was kindly donated by Paul Langford, Imperial College London (London, UK). Fragments containing each promoter followed by *gfp* were cloned into the pSET2 vector by using the ClonExpress MultiS one-step cloning kit (Vazyme, China), resulting in plasmids pSET2-P_g_*-gfp*, pSET2-P_t_*-gfp*, and pSET2-P_e_*-gfp*. The recombinant plasmids were transformed into S. suis by electroporation (2.5 kV, 25 mF, and 200 Ω). To construct ATc- and IPTG-inducible expression plasmids, the *tetR* gene and *lacI* gene were amplified from plasmids pRAB11 and pET-28a, respectively (37). The promoter fragments with the operator sequence inserted were synthesized by Sangon Biotech Company (Shanghai, China).

### Inducible expression of GFP.

S. suis cells cultured overnight were transferred to 4 mL of TSB and grown at 37°C to the mid-log phase (OD_600_ of ~0.5); 200 ng/mL ATc or 0.2 mM IPTG was then added, followed by another 1 h of incubation to induce expression. The cells were then harvested, washed three times with phosphate-buffered saline (PBS), resuspended in PBS, and diluted to give an OD_600_ of about 0.5. The suspension (200 μL) in four replicates was subjected to measurement of GFP fluorescence intensity in 96-well plates using a microplate reader (TECAN SPARK10M, Switzerland) (excitation at 485 nm and emission at 535 nm). S. suis strain SC19 was used as the negative control. The expression of GFP was further verified by Western blotting as follows. S. suis cells cultured overnight were transferred to 40 mL of TSB and grown under different culture conditions. The samples were collected by centrifugation, washed three times with PBS, and lysed with lysozyme treatment, followed by homogenization. The concentration of total protein was measured using a micro-bicinchoninic acid (BCA) protein assay kit (Cwbiotech, Beijing, China) and normalized to 15 mg for each sample. The samples were subjected to 12% SDS-PAGE analysis, followed by transfer to a polyvinylidene fluoride (PVDF) membrane by electrophoretic transfer. The GFP protein was probed with anti-GFP antibody (catalog number 50430-2-AP; Proteintech) (1:10,000 dilution) as the primary antibody and horseradish peroxidase (HRP)-conjugated goat anti-rabbit IgG (catalog number SA00001-2; Proteintech) (1:10,000 dilution) as the secondary antibody. The HylA protein as a control was detected with mouse anti-HylA serum (developed by our laboratory) (1:5,000 dilution) as the primary antibody and HRP-conjugated goat anti-mouse IgG (catalog number SA00001-1; Proteintech) (1:10,000 dilution) as the secondary antibody. Chemiluminescent signals of the protein bands were detected using a Western enhanced chemiluminescence (ECL) substrate kit (catalog number 1705060; Bio-Rad) and the ChemiDoc Touch imaging system (Bio-Rad).

### Determination of β-galactosidase activity.

In order to determine the optimal induction conditions, *lacZ* was used as a reporter gene; it was amplified by PCR amplification from the chromosomal DNA of E. coli BTH101 (45) and cloned into the inducible plasmids. S. suis cells cultured overnight were transferred to 80 mL of TSB and grown at 37°C to the mid-log phase (OD_600_ of ~0.5). The samples were then induced with the addition of ATc and IPTG, respectively, with different inducer concentrations and induction times. The samples were collected by centrifugation, washed three times with PBS, and resuspended with Z-buffer (46). The OD_600_ was recorded. The cell suspension was lysed with lysozyme treatment, followed by homogenization. *o*-Nitrophenyl-β-d-galactopyranoside (ONPG) was added to the cell lysate, followed by incubation at 37°C for 15 min, and the reaction was terminated by the addition of Na_2_CO_3_. The absorbance was measured at the wavelength of 420 nm and 550 nm, respectively. The β-galactosidase activity was calculated as 1,000 × (OD_420_ – [1.75 × OD_550_])/(*T* × *V* × OD_600_), in which *T* represents the reaction time and *V* represents the volume of the reaction (47).

### Construction of a *glmS* depletion/complementation strain.

In order to construct the *glmS* deletion strain of S. suis, the regions upstream and downstream of the *glmS* gene were amplified by PCR using primers GU-F/GU-R and GD-F/GD-R, respectively (Table 2). The PCR products were cloned into the Ts plasmid pSET4s via the BamHI site by using ClonExpress MultiS one-step cloning kit (Vazyme). The resulting plasmid was introduced into S. suis SC-19 by electroporation (2.5 kV, 25 mF, and 200 Ω). The transformants were grown in TSB with 10 mM GlcNAc. The single- and double-exchanged strains were screened as described previously (8) using culture medium supplemented with GlcNAc. The *glmS* deletion strain was verified by PCR using primers Rglms-F and Glms-R. To construct the induced complementary strain, the *glmS* gene was amplified by PCR using primers Rglms-F and Glms-R and fused to an ATc-inducible promoter amplified by PCR from plasmid pSSTete2-*gfp*. The PCR products were cloned into plasmid pSET2 via the BamHI site, and the resulting plasmid was then transformed into the *glmS* deletion strain to acquire the induced complementary strain.

**TABLE 2:** Primers used in the present study

Primer name	Primer sequence
P_g_-F	5′-TGCAGGTCGACTCTAGAGGATCCTAAGATGAACCGGTAAGCAG-3′
P_g_-R	5′-CTTTACTCATGAATGATTTCCTCCTTATGAA-3′
gGFP-F	5′-GAAATCATTCATGAGTAAAGGAGAAGAAC-3′
GFP-R	5′-CGAGCTCGGTACCCGGGGATCCCTATTTGTATAGTTCATCCATG-3′
P_t_-F	5′-TGCAGGTCGACTCTAGAGGATCCTATAAGCGAAGCTAATAGCCC-3′
P_t_-R	5′-CTTTACTCATTTTGGTAAAAGCCTCCAATAA-3′
tGFP-F	5′-TTTTACCAAAATGAGTAAAGGAGAAGAAC-3′
P_e_-F	5′-TGCAGGTCGACTCTAGAGGATCCAAAAGGAAGGCGTTTACATTAT-3′
P_e_-R	5′-CTTTACTCATTATATTACTCTCCTTTGAGTTT-3′
eGFP-F	5′-GAGTAATATAATGAGTAAAGGAGAAGAAC-3′
tetR-F	5′-TGCAGGTCGACTCTAGAGGATCCTTAAGACCCACTTTCACATTT-3′
tetR-R	5′-GAAATCATTCATGATGTCTAGATTAGATAAAAG-3′
P1-F	5′-TAGACATCATGAATGATTTCCTCCTTATGAA-3′
P1-R	5′-CTTTACTCATTTTGGTAAAAGCCTCCAATAAA-3′
1GFP-F	5′-TTTTACCAAAATGAGTAAAGGAGAAGAAC-3′
P2-R	5′-CTTTACTCATTATATTACTCTCCTTTGAGTTT-3′
lacI-F	5′-TGCAGGTCGACTCTAGAGGATCCTCACTGCCCGCTTTCCAG-3′
lacI-R	5′-GAAATCATTCGTGAAACCAGTAACGTTATAC-3′
lacR-F	5′-CTGGTTTCACGAATGATTTCCTCCTTATGAAA-3′
tetRlzD-R	5′-TCATGGTCATTATATTACTCTCCTTTGAGTTT-3′
lacZ-F	5′-GAGTAATATAATGACCATGATTACGGATTC-3′
lacZ-R	5′-CGAGCTCGGTACCCGGGGATCCTATTTTTGACACCAGACCAAC-3′
Pz-R	5′-AAAATGCCATTATATTACTCTCCTTTGAGTTT-3′
FtsZ-F	5′-GAGTAATATAATGGCATTTTCATTTGAAGCA-3′
FtsZ-R	5′-CAGGAACTCGATGTCTAGTTTGCGATTACGGAAGAATGGT-3′
zGFP-F	5′-CTAGACATCGAGTTCCTGCAGATGAGTAAAGGAGAAGAAC-3′
GU-F	5′-TGCAGGTCGACTCTAGAGGACATCCCGCCTTACGTACCA-3′
GU-R	5′-CCTTATCTTCACATATCACCTACGATGTTTG-3′
GD-F	5′-GGTGATATGTGAAGATAAGGTTCGGATTTC-3′
GD-R	5′-CGAGCTCGGTACCCGGGGATCCTGGCAGTTCTTGACCGTTAT-3′
tetRg-R	5′-TTCCACACATTATATTACTCTCCTTTGAGTTT-3′
Rglms-F	5′-GAGTAATATAATGTGTGGAATCGTTGGTG-3′
Glms-R	5′-CGAGCTCGGTACCCGGGGATCCTTATTCAACAGTAACAGACTTA-3′

### Fluorescence microscopy.

The S. suis cells were grown in 5 mL of TSB at 37°C until the OD_600_ reached 0.3. The cells were transferred into 5 mL of fresh TSB with a ratio of 50:1 and grown for 0.5 h. To induce protein expression, ATc was supplemented at 100 ng/mL, followed by incubation at 37°C for 15 min. The cells were collected by centrifugation, washed with PBS three times, and resuspended in 500 μL of PBS. Fluorescent dye AF647 (catalog number A20006; Thermo Fisher Scientific) was then added to a final concentration of 20 μg/mL, followed by incubation at 37°C for 45 min. Twenty microliters of the sample was spotted on a microscope cover glass and covered with a 1% agarose pad. Images were collected by using the NIS-Elements microscope (Nikon, Japan) and analyzed by using ImageJ software. For FtsZ-GFP imaging, a 100-ms exposure time, excitation at 488 nm, and emission at 500 to 545 nm were used. For AF647 imaging, a 20-ms exposure time, excitation at 647 nm, and emission at 663 to 738 nm were used.

### Data availability.

The sequences of plasmids pSSTete2-*gfp* and pSSlace1-*gfp* are available in the NCBI GenBank database with the accession numbers ON391044 and ON391045, respectively.
